# Ferroptosis: Shedding Light on Mechanisms and Therapeutic Opportunities in Liver Diseases

**DOI:** 10.3390/cells11203301

**Published:** 2022-10-20

**Authors:** Hongmei You, Ling Wang, Fangtian Bu, Hongwu Meng, Cheng Huang, Guoying Fang, Jun Li

**Affiliations:** 1 Department of Pharmacy, Hangzhou Women’s Hospital, Hangzhou 310000, China; 2 Department of Pharmacy, Shangyu People’s Hospital of Shaoxing, Shaoxing 312000, China; 3 Inflammation and Immune Mediated Diseases Laboratory of Anhui Province, Anhui Institute of Innovative Drugs, School of Pharmacy, Anhui Medical University, Hefei 230000, China

**Keywords:** regulated cell death, ferroptosis, liver diseases

## Abstract

Cell death is a vital physiological or pathological phenomenon in the development process of the organism. Ferroptosis is a kind of newly-discovered regulated cell death (RCD), which is different from other RCD patterns, such as apoptosis, necrosis and autophagy at the morphological, biochemical and genetic levels. It is a kind of iron-dependent mode of death mediated by lipid peroxides and lipid reactive oxygen species aggregation. Noteworthily, the number of studies focused on ferroptosis has been increasing exponentially since ferroptosis was first found in 2012. The liver is the organ that stores the most iron in the human body. Recently, it was frequently found that there are different degrees of iron metabolism disorder and lipid peroxidation and other ferroptosis characteristics in various liver diseases. Numerous investigators have discovered that the progression of various liver diseases can be affected via the regulation of ferroptosis, which may provide a potential therapeutic strategy for clinical hepatic diseases. This review aims to summarize the mechanism and update research progress of ferroptosis, so as to provide novel promising directions for the treatment of liver diseases.

## 1. Introduction

Liver disease with high morbidity and mortality accounts for the heavy burden of disease and costs worldwide. Statistically, about 2 million people around the world die of liver diseases [1]. Cell death plays a pleiotropic role in the development and process of various forms of liver diseases. Unlike accidental cell death triggered by serious mechanical, physical and chemical injury, regulated cell death (RCD) can be controlled pharmacologically and genetically. Thus, it is under the regulation of specific intracellular mechanisms [2]. A better understanding of the molecular mechanisms governing cell death is vital for confirming its role in various diseases. The first found form of RCD at the molecular level was caspase-dependent apoptosis. In recent years, necroptosis, autophagy, pyroptosis, and other forms of RCD have been found and explored. Notably, ferroptosis, a newly discovered form of RCD, found by Dixon and his team in 2012 [3], is considered as one of the most common and oldest forms of cell death. Ferroptosis-like cell death was not only observed in mammals, but also in remote species, such as plants, fungi and protozoa [4,5,6], etc. Over recent years, the field of ferroptosis research in liver diseases has been increased exponentially, since the concept “ferroptosis” was created in 2012.

Ferroptosis is an iron-dependent form of RCD, which is mediated by high levels of lipid peroxides and lipid reactive oxygen species (ROS) [7]. It is different from other types of RCD in cell morphology and biochemical characteristics. In morphological aspect, ferroptotic cells mainly show mitochondria shrinkage with accessorial membrane density, reduction or disappearance of mitochondrial crista, and the rupture of outer mitochondrial membrane, which are absolutely different features from other modes of RCD [7,8]. In contrast to apoptosis, ferroptosis does not show cell shrinkage, condensation of chromatin, apoptotic body formation and cytoskeleton disintegration. Compared to autophagy, ferroptosis lacks classical autophagic vacuoles, which is a kind of closed bilayer membrane structure. Unlike necrosis, ferroptosis does not exhibit cytoplasm and organelles swelling and cell membrane rupture. In biochemical aspects, ferroptotic cells show a variety of biological processes and chemical changes, including but not limited to iron, amino acid and polyunsaturated fatty acid, glutathione biosynthesis, GPX4, FSP1, GCH1, NADPH, and coenzyme Q10, etc. [9].

The liver is a pivotal organ for regulating various physiological metabolic processes, such as the metabolism of glucose, lipids and amino acids. Abnormal expression of these elements can result in oxidative stress or severe liver disease [10,11]. An increasing number of studies have shown that hepatic metabolic pathways are closely related to ferroptosis [12,13,14,15]. After dietary intake, glucose will enter the liver rapidly and sequester as glycogen. Glucose can generate energy through glycolysis and generate NADPH. The metabolic disorder of essential fatty acids is vitally important, since the excessive accumulation of lipid peroxidation is regarded as one of the critical characteristics of ferroptosis [16]. With respect to amino acids, some amino acids are directly associated with ferroptosis via regulating oxidative stress. Amino acids cannot transport nutrients such as sugar into cells directly. They must be diffused across cell membrane with the aid of specific transporters, such as system Xc−, which is comprised of SLC3A2 and SLC7A11. System Xc− accelerates the exchange of cystine and glutamate across the cell plasma membrane. Once transported into the cell, cystine will be reduced to cysteine, which is the limiting amino acid in GSH synthesis. Reduced GSH is the essential intracellular antioxidant in mammals and is generated from glycine, glutamate and cysteine. Thus, the biosynthesis of GSH is influenced by cystine and cysteine availability [17]. Inhibiting the import of cystine through system Xc− is sufficient to trigger ferroptosis via depleting GSH levels [18]. Of note, an increasing number of studies have shown that cystine deprivation can induce ferroptosis in a variety of diseases [19,20,21,22,23].

There are about 2 million people dying of different liver diseases per year worldwide. Some of them are due to the complications of cirrhosis, and others are due to acute or chronic hepatitis and hepatocellular carcinoma [1,24]. The liver is the most important organ for storing iron in the human body. A growing number of research studies indicated that ferroptosis plays an essential role in the progression of various forms of liver diseases, such as alcohol liver disease, virus hepatitis, non-alcohol steatohepatitis, liver fibrosis, hepatocellular carcinoma, etc. [25,26]. In this review, we summarize the recent advance of ferroptosis in liver diseases, proposing the possibility of explaining how ferroptosis cures or leads to diseases. We also discussed the possibility of treating clinical liver diseases through targeting ferroptosis-related genes.

## 2. Mechanisms Regulating Ferroptosis

**Figure 1:**
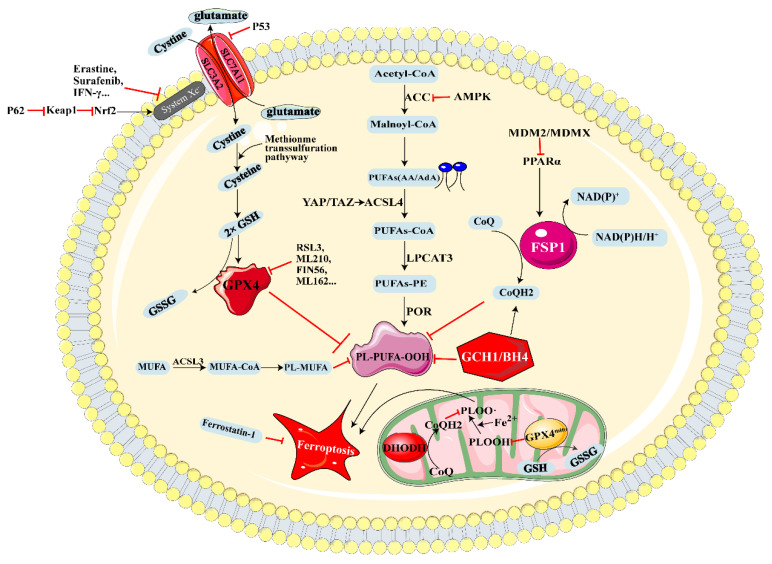
Summary of core regulators and main mechanisms of ferroptosis. The major systems regulating ferroptosis include GPX4-related ferroptosis defense, FSP1-associated system, GCH1/BH4-related system and DHODH-mediated ferroptosis resistance. The core components and regulators involved in the regulation of ferroptosis are exhibited and the detailed description is described in the main text. Abbreviations: ACSL4: acyl-CoA synthetase long-chain family member 4; BH4: Tetrahydrobiopterin; CoQ: oxidized coenzyme Q; CoQH2: reduced coenzyme Q; GCH1: GTP Cyclohydrolase1; GPX4: glutathione peroxidase 4; GSH: reduced glutathione; GSSG: oxidized glutathione; IFN-γ: Interferon γ; LPCAT3: lysophosphatidylcholine acyltransferase 3; MDM2: Murine double minute 2; MDMX: Murine double minute X; MUFA: monounsaturated fatty acid; NAD(P)^+^: oxidized nicotinamide adenine dinucleotide (phosphate); NAD(P)H: reduced nicotinamide adenine dinucleotide (phosphate); Nrf2: nuclear factor erythroid 2-related factor2; PLOO·: phospholipid hydroperoxyl radical; PLOOH: phospholipid hydroperoxyl; PL-PUFA-OOH: polyunsaturated fatty acid-phospholipid ethanolamine hydroperoxide. The black arrow means to accelerate the process. The red T bar means to inhibit the process.

As mentioned above, ferroptosis is a type of RCD, characterized by excessive lipid hydroperoxides caused by iron overload. Since ferroptosis, this newly discovered form of cell death was first reported in 2012 by Dixon et al. [3], and the mechanisms governing ferroptosis were gradually clearly explored, mainly focusing on the metabolism of cysteine and glutathione, GPX4 (the phospholipid peroxidase) [27], FSP1 (ferroptosis suppressor protein 1) [14] and GCH1/BH4 [28], etc. (Figure 1). Unrestrained peroxidation of phospholipids is the central part of ferroptosis. Phospholipids with polyunsaturated acyl tails (PUFA-PLs), because of their susceptibility to peroxidation chemistry, are the main substrate for peroxidation during the process of ferroptosis. Consistent with this, based on CRISPR/Cas9 and genome-wide haploid screening, two portal drivers of ferroptosis, acyl-CoA synthetase long-chain family member 4 (ACSL4) and lysophosphatidylcholine acyltransferase 3 (LPCAT3), were found, which could generate PUFA-PLs by activating and incorporating free PUFAs into phospholipids [29,30,31]. Thus, controlling the content of PUFA-PLs, the inducers of peroxidation and the factors that inhibit lipid peroxides are all strategies to regulate ferroptosis.

### 2.1. GPX4-Regulated Ferroptosis

In the early 1980s, Ursini and colleagues originally discovered glutathione peroxidase 4 (GPX4, a selenoprotein) through biochemical purification, and identified its role in catalyzing PLOOH to PLOH in mammalian cells [32,33]. As one of 25 specific selenoproteins in humans, the expression of GPX4 is precisely regulated [34] and GPX4 is the major PLOOH-neutralizing enzyme. It is worth noting that GSH, the richest reductant in mammalian cells, is the essential cofactor for glutathione peroxidase (GPX). GSH can be generated from cysteine that is generated from methionine by trans-sulfuration reaction [35], or from system Xc−, which pumps glutamate out and cystine into cells. Furthermore, cystine can be mutated into cysteine through redox reactions [2]. Previous research has shown that cyst(e)ine-deficient cells would die in a short time but increasing the synthesis of endogenous cysteine with methionine and glucose would reduce this kind of cell death [36,37]. System Xc− cystine/glutamate antiporter, composed by subunits SLC71A11 and SLC3A2, is the central part for the regulation of ferroptosis [38,39]. Further mechanistic investigation revealed that Erastin, RSL3 and ML210 promote ferroptosis by inhibiting system Xc− and GPX4, respectively [27,40,41,42]. Additionally, Wang and colleagues discovered IFN-γ released from CD8+ T cells could enhance ferroptosis in tumor cells by downregulating the expression of SLC3A2 and SLC7A11 [43]. In contrast, p62-Keap1-NRF2 was reported to diminish ferroptosis in cancer cells by upregulating SLC7A11 and increasing the secretion of glutamate [44].

### 2.2. FSP1-Regulated Ferroptosis

FSP1 (ferroptosis-suppressor-protein 1, formerly known as AIFM2) has the ability to inhibit ferroptosis, which was initially found through expression cloning technology in GPX4-deficient cells. Additionally, the ferroptosis-resistant function of FSP1 is independent of GSH content, GPX4 activity and ACSL4 levels [12,14]. Thus, FSP1 became the second major anti-ferroptosis mechanism after the cyst(e)ine–GSH–GPX4 axis. Mechanically, FSP1 acts as an oxidoreductase to convert ubiquinone (CoQ) to ubiquinol (CoQH2). CoQH2 functions as a lipophilic radical-trapping antioxidant to clear lipid peroxyl radicals [45]. Moreover, Verkatesh et al. discovered FSP1 can be activated by PPARα, which is directed by the MDM2.MDMX complex [46].

### 2.3. GCH1-Regulated Ferroptosis 

GTP cyclohydrolase-1 (GCH1) and its antioxidant metabolites tetrahydrobiopterin/dihydrobiopterin (BH4/BH2) were found to have antagonistic properties against ferroptosis by genome-wide activation screen technology [28]. Mechanistically, a high expression of GCH1 and BH4/BH2 could lead to lipid remodeling, prevent the depletion of phospholipids with two polyunsaturated fatty acyl tails, and ultimately inhibit ferroptosis. In addition, the GCH1–BH4–phospholipid axis could also suppress the formation of PL–PUFA–OOH through regulating the abundance of CoQ [28]. 

### 2.4. Other Mechanisms Regulating Ferroptosis Sensitivity

After GPX4 and FSP1, the two major mechanisms against ferroptosis were discovered; Mao et al. [45] found supplementation with the product orotate and substrate dihydroorotate of dihydroorotate dehydrogenase (DHODH) could enhance or attenuate ferroptosis in GPX4^low^ cancer cells. Mechanistically, the DHODH gene could encode dihydroorotate dehydrogenase, which is located on the outer surface of mitochondrial intima and involved in pyrimidine synthesis. Furthermore, DHODH works synergistically with mitochondrial GPX4 to suppress ferroptosis via reducing ubiquinone to ubiquinol. 

What is more, while monounsaturated fatty acids (MUFA) are activated by ACSL3, the sensitivity of phospholipids to lethal oxidation can be reduced significantly in a short time [47]. Consequently, the process of ferroptosis is suppressed. Additionally, AMPK could inhibit ferroptosis by inhibiting ACC [48]. Furthermore, antagonizing the Merlin–Hippo signalling axis triggers the activity of YAP to induce ferroptosis by upregulation of ACSL4 and transferrin receptor [49]. Transcription factor p53 can decrease the uptake of cystine through inhibiting SLC7A11 expression, and ultimately increase cellular sensitivity to ferroptosis [50].

## 3. Links between Ferroptosis and Liver Diseases

Structurally, the liver is composed of parenchymal cells (hepatocytes) and nonparenchymal cells. Hepatocytes play a critical role in maintaining stable glucose and lipoprotein content in the plasma. Hepatocyte death occurs in almost every type of liver disease and is viewed as a major index for the examination of various acute and chronic hepatic diseases of toxic, viral, alcohol, metabolic or autoimmune origin [51]. Liver sinusoidal endothelial cells, hepatic macrophages and hepatic stellate cells (HSCs) constitute the liver microcirculatory milieu and control liver homeostasis. Macrophages are extensively involved in the initiation and development of a variety of liver diseases [52,53]. HSCs account for 5–8% of cells and store lots of vitamin A lipid droplets in a healthy liver [54,55]. When the liver is damaged by toxins, viral infection or other injury factors, HSCs can be activated and become proliferative, contractile and fibrogenic, serving as the major ECM producer in the liver, which is the main pathological feature of liver fibrosis [56]. Noteworthily, the occurrence and development of liver diseases are the result of crosstalk among different types of cells. Fortunately, increasing studies have shown that the progression of liver disease can be moderated by targeting the cells [57,58,59,60]. Iron overload and oxidative stress are the two most important causes of liver damage and disease development in most hepatopathies [61,62,63]. Ferroptosis, characterized by iron overloading and lipid peroxidation, has been widely reported to regulate multiple liver diseases by targeting the cells mentioned above. The following review will concentrate on summarizing the proven function of ferroptosis in a wide spectrum of hepatic diseases.

### 3.1. Ferroptosis and Acute Liver Injury

Acute liver injury (ALI) is a rare but life-threatening critical disease characterized by a rapid reduction in hepatocyte function that occurs most frequently in individuals without pre-existing liver disease [64,65]. Drugs, hepatic ischemia reperfusion injury (IRI), alcohol and viral hepatitis are common pathogenic factors of ALI [65]. Among the drugs with hepatotoxicity, the typical nonsteroidal anti-inflammatory drug paracetamol (N-acetyl-para-aminophenol, or APAP) is the leading cause of ALI [66].

#### 3.1.1. Ferroptosis and APAP-Induced Acute Liver Injury

APAP-induced ALI often occurs after intentional overdose or unintentional ingestion (therapeutic error) and APAP-induced hepatoxicity is dose-dependent [66]. After APAP at the therapeutic dose enters the liver, approximately 85–90% of APAP undergoes a phase II metabolic reaction with the participation of UDP-glucuronyltransferase (UGTs) and sulfotransferase (SULTs) and transforms to non-toxic compounds, which are then excreted in urine [67,68]. About 2% of APAP is excreted in prototype form with urine [69]. The remaining 10% of APAP undergoes phase I oxidation with cytochrome P450 2E1 (CYP2E1) and is converted into a highly reactive and toxic intermediate, N-acetyl-para-benzoquinoneimine (NAPQI), which will combine with GSH to form non-toxic compounds and be excreted in urine [68,69,70]. Excessive APAP can cause the production of NAPQI, which will bind to GSH, further leading to the depletion of GSH in hepatocytes. Thus, GSH decline, iron overload and lipid peroxidation have been considered as the basic mechanisms of APAP-caused liver injury [71]. Increasing numbers of researchers have verified that ferroptosis is involved in APAP-induced cell death [72,73]. Lőrincz et al. [72] found that in addition to necroptosis and apoptosis, ferroptosis is also involved in APAP-induced hepatocyte death. Subsequently, Yamada et al. [26] reported that ferroptosis mediates the hepatotoxicity and mortality that APAP induces in a murine model. The ferroptosis inhibitor Ferrostatin (Fer-1), α-tocopherol and the iron chelator deferoxamine could effectively suppress APAP-induced hepatic injury, lipid peroxidation and GSH exhaustion. Mass spectrometry and gene inhibition of ACSL4 further revealed that radical oxidation of n-6 polyunsaturated fatty acids (mainly arachidonic acid) contributed to APAP-induced ferroptosis in hepatocytes. What is more, Wang et al. [74]. have verified that ulinastatin could reduce APAP-induced liver injury via suppressing ferroptosis through the SIRT1/Nrf2/HO-1 pathway.

In addition, some compounds, such as 3,4-dihydroxyphenylethyl alcohol glycoside isolated from sargentodoxa cuneata, have been proven to inhibit ferroptosis in APAP-induced liver injury [75]. In conclusion, ferroptosis may play an important role in APAP-induced ALI and a better understanding of the regulatory role of ferroptosis may provide a potential clinical therapeutic approach.

#### 3.1.2. Ferroptosis and Ischemia-Reperfusion Injury (IRI)

IRI is the result of revascularization following a temporary reduction in blood supply [76], which will aggravate pre-existing disease in the liver. Liver IRI is still a tough clinical problem, which can be induced by liver transplantation, hepatic resection surgery and shock (such as sepsis and hemorrhage). Moreover, liver IRI can be divided into two phases: ischemia and reperfusion. These two stages are the consequence of oxidative stress accompanied by loss of blood flow, nutrition deficiency, inflammation and other mechanisms [77]. During reperfusion, iron-mediated death is thought to be related to oxidative stress from ROS [78,79]. What is more, ferroptosis is a form of iron-dependent cell death characterized by excessive accumulation of ROS. Friedmann Angeli et al. [80] reported that knockout of GPX4 in cells results in severe ferroptosis pathologically, and Liprostatin-1, a kind of ferroptosis inhibitor, can suppress ferroptosis significantly in GPX4^−/−^ mice. Further pre-clinical trials show that kidney IRI and liver IRI are obviously decreased by Liprostatin-1, indicating that ferroptosis plays a significant role in IRI [80]. Wu et al. [53] found that targeting macrophages and inhibiting the release of macrophage extracellular traps (METs) could prevent iron overload and further inhibit hepatocyte ferroptosis, and eventually protect liver from damage caused by IRI. What is more, liver IRI can be significantly prevented by ferrostatin-1 and α-tocopherol or by an iron chelator such as deferoxamine [81]. Collectively, these studies demonstrated that ferroptosis is an innovative hazardous factor for liver IRI, and inhibition of ferroptosis may effectively relieve the symptoms of IRI.

### 3.2. Ferroptosis and Chronic Liver Injury

At present, chronic liver injury (CLI) is a kind of refractory public disease that impairs human health, with annual increasing incidence [82,83]. When the body is exposed to alcohol, harmful chemical materials such as CCl_4_, chronic infection viruses such as HBV/HCV or intermediary metabolism, the liver is the most susceptible and vulnerable organ with the occurrence of CLI [84,85]. The persistence of damaged elements will result in the excessive accumulation of lipids and peroxidation, which will further lead to chronic inflammation and steatohepatitis [86,87]. Then, the over-deposition of extracellular matrix (ECM) can disrupt the normal physiological architecture of the liver [55]. In the early stages, once the intervention is not performed in a timely manner, CLI will develop into hepatitis, fibrosis, cirrhosis and even hepatocellular carcinoma [88]. Recently, an increasing number of studies have demonstrated the function of ferroptosis in CLI. This will be described in detail in the following text. 

#### 3.2.1. Ferroptosis and Virus Hepatitis

Virus hepatitis is the leading cause of CLI, and chronic HCV infection promotes iron accumulation, upregulating duodenal ferroportin-1 and downregulating hepatic hepcidin, which is the master regulator of systemic iron homeostasis [89,90]. In hepatocytes, iron is stored in ferritin and exported by ferroportin. Hepcidin suppresses intracellular iron efflux via binding to and facilitating the degradation of ferroportin. Excessive iron accumulation directly correlates with the worsening of hepatic damage. Currently, whether ferroptosis plays an effective role in the development of HBV/HCV remains to be further explored. The study conducted by Yamane et al. [91]. showed that HCV replication was suppressed by ferroptosis and mediated by the non-classical desaturation of oleate to mead acid and other unsaturated fatty acids by fatty acid desaturase 2 (FADS2), which is the critical determinant of cell sensitivity to ferroptosis. Thus, inhibition of FADS2 significantly suppresses ferroptosis and further markedly enhances the replication of HCV [91]. Conversely, Erastin, the ferroptosis-inducing compound, changes the construction of HCV replicases and further sensitizes it to anti-virus agents targeting the viral protease [91]. What is more, Zhang et al. [92]. reported that the exosomes secreted by HBV-infected hepatocytes aggravate hepatic fibrosis through the miR-222/TFRC axis. Altogether, the above studies show that ferroptosis may serve as a new therapeutic strategy for the virus hepatitis.

#### 3.2.2. Ferroptosis and Non-Alcoholic Fatty Liver Disease

Non-alcoholic fatty liver disease (NAFLD) is a kind of CLI that spans from simple steatosis to non-alcoholic steatohepatitis (NASH) and is assessed to influence up to one-third of the total population globally [93]. The development of NAFLD is closely related to obesity and metabolic syndrome [94]. Recently, Gautheron and his team proposed an iron-centered hypothesis during the progression of NAFLD [94]. It is widely known that excessive iron accumulation is a common index in patients with NAFLD, and lipid peroxide induced by iron is one of the prime contributors to NAFLD [95,96]. What is more, iron overload is closely related to insulin resistance and obesity, two representative traits of NAFLD patients [97]. Tsurusaki et al. [98]. verified that ferroptosis, but not necroptosis, is the major cell death to initiate inflammation and cell death in NASH, and the ferroptosis inhibitors (such as trolox or deferoxamine) can strongly reverse hepatocyte death, inflammation and lipid peroxidation during the initial phase of NASH. Iron overload triggered by metabolic dysfunction (such as hereditary hemochromatosis and liver ironosis) can accelerate the progression of NASH [99,100]. In addition, ferroptosis inhibitor (such as ferrostatin-1), and iron removal (with deferoxamine mesylate salt) can effectively improve liver injury in NAFLD [101,102,103,104]. In addition to iron accumulation, MDA and 4-HNE (two lipid peroxidation markers) are also increased in NASH patients [105]. The lipid peroxidation and serum transaminases are suppressed by Vitamin E in NASH patients [106]. All these studies indicate that ferroptosis induced by iron accumulation and lipid peroxidation might be associated with the pathological progression of NAFLD and that inhibition of ferroptosis might provide a novel therapeutic strategy for NAFLD.

Further mechanistic studies have found that GPX4 can slow down NASH progression via inhibiting ferroptosis, and RSL-3, an inhibitor of GPX4, aggravated the liver image of NASH by facilitating ferroptosis in MCD diet-fed mice. Conversely, sodium selenite, an activator of GPX4, improved the pathologic characteristics of NASH [102]. Consistently, liver cell death is also influenced by GPX4-induced ferroptosis in a palmitic acid-induced NASH model in vitro [107]. What is more, Thymosin β4 [103] and Enolase 3 [108] were reported to regulate the progression of NASH via inhibiting or increasing GPX4 expression. Wei et al. found that the expression of ACSL4 was elevated in arsenic-induced NASH models in vivo and in vitro. In addition, ACSL4 inhibition with rosiglitazone or specific siRNA can significantly suppress ferroptosis and further alleviate the progression of arsenic-induced NASH via restraining 5-HETE content [109]. Moreover, Nrf2 was also reported to regulate the synthesis of fatty acids by inhibiting enzyme expression and suppress NASH progression via the p62–Keap1–Nrf2 axis [110]. Additionally, Nrf2 plays an essential role in antioxidation response and alleviating ferroptosis. Notably, activation of the Nrf2 pathway can decrease liver lipid deposition and effectively improve the pathology of NAFLD in vivo [111]. Dehydroabietic acid [95] and Ginkgolide B [111] improve NAFLD via inhibiting ferroptosis through upregulating Nrf2 and its downstream genes (such as HO-1, GPX4, etc.). In addition, other mechanisms, such as ECH1 and miR-33, have also been reported to participate in the progression of NAFLD via regulating ferroptosis [112,113]. Altogether, studies of ferroptosis in NAFLD need further exploration in relevant animal models and in patients, especially since no accurate choices are available for NAFLD treatment at present. 

#### 3.2.3. Ferroptosis and Hepatic Fibrosis

Physiologically, hepatic stellate cells (HSCs) are quiescent and rich in vitamin A lipid droplets [54]. When the liver is injured by multi-stimuli such as toxins or chemical material, quiescent HSCs will differentiate into activated contractile, proliferative and fibrogenic HSCs, which are the leading target for the treatment of liver fibrosis [56]. Overexpression of TRIM26 inhibits HSCs proliferation, accelerates HSCs ferroptosis and further alleviates CCl4-induced liver fibrosis via interacting with SLC7A11 and promoting its ubiquitination [114]. In addition, many other compounds or plant extracts (such as magnesium isoglycyrrhizinate [115], artemether [116], artesunate [117], and Sorafenib [118], etc.) are able to target activated HSCs, trigger their ferroptosis and eventually slow down the progression of liver fibrosis. Moreover, ZFP36 [119], IRP2 [120], ELAVL1/HuR [121], and the BRD7–P53–SLC25A28 axis [122], etc. are all regulatory factors involved in the ferroptosis of HSCs. What is more, epigenetic m^6^A modification [123] was also reported to be associated with the process of ferroptosis in HSCs. Altogether, these results manifest that regulating ferroptosis by targeting HSCs may be a new therapeutic approach for treating liver fibrosis in the clinic. A growing number of studies indicate that superfluous iron is relevant to liver injury via ferroptosis [3,124]. Transferrin (Trf) is responsible for transferring Fe^3+^ to other organs or bone marrow. When the level of Trf decreases, excessive non-Trf-bound-iron (NTBI) will increase in hepatocytes. Further study showed that when Trf-KO mice were treated with ferrostatin, a ferroptosis inhibitor, the symptoms of liver fibrosis induced by either CCl4 injection or high dietary iron were rescued potently [125]. In addition, combined with the clinical data, we can conclude that Trf plays a protective role in liver fibrosis via suppressing ferroptosis [125]. 

#### 3.2.4. Ferroptosis and Hepatocellular Carcinoma

In economically developed and developing countries, carcinoma is respectively the leading and second leading cause of cancer-associated death [126,127]. Among the primary hepatic cancers, hepatocellular carcinoma (HCC) takes up 70–85% of the total burden of liver cancer globally, representing the main histological subtype [128]. Surgical resection and conservative non-surgical treatment are both therapeutic strategies for advanced HCC, but both are of restricted treatment effect. Sorafenib, as the first and only approved first-line drug for advanced HCC, improves the survival rate of patients to some extent [129,130]. However, emerging drug resistance and severe adverse reactions to Sorafenib are the leading causes of poor clinical prognosis. Interestingly, a large number of researchers have manifested that Sorafenib can induce ferroptosis via inhibiting SLC7A11 [40,131,132], blocking oxidized cysteine from entering cells. Excessive intake of dietary iron can raise the risk of HCC, indicating that excessive iron content can accelerate the progression of HCC [133]. In addition, a growing number of pathways and mechanisms have been found to regulate Sorafenib resistance and the progression of HCC via aiming at ferroptosis [25,134]. Collectively, Sorafenib-induced ferroptosis may be an effective and promising strategy for the induction of cancer cell death in HCC. In this review, we will summarize recent studies on the relationship between HCC and ferroptosis, and further evaluate the possibility of ferroptosis as a therapeutic target for HCC. 

In recent years, more and more studies have clarified the essential role of ferroptosis in HCC. Many genes, such as retinoblastoma (Rb) protein [135], Nrf2 [25], metallothionein-1G (MT-1G) [136], CDGSH iron sulfur domain 1 (CISD1, also named mitoNEET) [137], TP53 gene (S47 variant) [138], etc. are known as the negative regulators of ferroptosis. Louandre et al. [135] reported that the expression and function of Rb protein is reduced during liver carcinogenesis. Further examination showed that Rb-negative HCC cells are more sensitive to death induced by Sorafenib compared with Rb-expressing HCC cells. The transcription factor Nrf2 was originally considered as a pivotal regulator of antioxidant intercellularly and was negatively controlled by Keap1 [139]. Overexpression of Nrf2 induces apoptosis and caused chemoresistance in lung carcinoma, breast adenocarcinoma and nephroblastoma [140]. Moreover, Sun et al. [25] reported that the p62–Keap1–Nrf2 pathway plays a pivotal role in protecting HCC cells from ferroptosis induced by ferroptosis-inducing compounds (such as Sorafenib, Erastin, and buthionine sulfoximine) via increasing the expression of several Nrf2-targeting genes (NQO1, HO1, FTH1) associated with ROS and iron metabolism. The anticancer effect of Sorafenib and Erastin in HCC cells increased markedly while the expression of Nrf2 was inhibited in vivo and in vitro. When these Nrf2-targeted genes were knocked down, the growth inhibition of ferroptotic inducer-treated HCC cells was significantly increased [25]. Another gene that negatively regulates Sorafenib-induced ferroptosis is MT-1G, which has been a prospective therapeutic target for improving Sorafenib resistance in HCC cells [136]. In addition, CISD1, an iron-containing outer mitochondrial membrane protein, negatively regulates ferroptosis in HCC cells. The genetic knockdown of CISD1 enhances iron-mediated lipid peroxidation, resulting in Erastin-induced ferroptosis in human HCC cells, such as HepG2 and Hep3B [137]. What is more, Jennies et al. [138]. found that when TP53 gene mutated into mutant p53 with Pro47 (S47 variant), it exhibited a slight decrease in apoptosis induced by genotoxic stress but showed significant resistance to ferroptosis induced by Erastin. What is more, compared to WT p53 HCC cells, after cisplatin stimulus, the S47 variant cells showed a higher expression level of SLC7A11 and lower PTGS content (a biomarker of ferroptosis in vivo), which manifested the resistance to ferroptosis [138]. Haloperidol was reported to promote both Sorafenib and Erastin-induced ferroptosis at a comparatively low dose, which would benefit HCC patients treated with Sorafenib by decreasing the dosage or enhancing its curative effect [141]. The traits of ferroptosis such as excessive iron accumulation, GSH depletion and lipid peroxidation were detected following haloperidol administration. Meanwhile, Nrf2, HO-1, GPX4 and other ferroptosis-associated targets were influenced by haloperidol [141]. Altogether, the above studies provide an innovative strategy for the treatment of HCC, that is, inducing ferroptosis of HCC cells may act as a novel and promising approach for HCC. 

The pivotal regulator of lipid peroxidation in ferroptosis needs further exploration, though that lipid peroxidation has as an essential role in promoting ferroptosis is known. Lipoxygenase, an iron-containing enzyme, is the central inducer of ferroptosis via generating lipid hydroperoxides, and its effect depends on the excitation of ACSL4-dependent lipid biosynthesis [142]. Feng and colleagues discovered that the expression of ACSL-4 can forecast sensitivity to Sorafenib in a series of HCC cell lines. ACSL4 inhibition by pharmacological methods and specific siRNA/sgRNA could significantly suppress Sorafenib-induced lipid peroxidation and ferroptotic cell death in Huh7 cells and rescue xenograft growth suppression in vivo [143]. In addition, low-density lipoprotein-docosahexaenoic acid (LDL-DHA) can also induce ferroptosis and further kill HCC cells, accompanied by GSH depletion and GPX4 inactivation [144]. Moreover, LDL-DHA nanoparticles were reported to lessen intracellular GSH via downregulating GSH/GSSG and NADPH/NADP^+^ (redox couples) and reducing GSH-aldehyde adducts in HCC cells [145]. Then, due to the elimination of substrate, GPX4 failed to function within its enzymatic activity. Recently, IFN-γ [146], YAP/TAZ and ATF4 [134], LIFR–NF–κB–LCN2 axis [147], lncRNA GABPB1-AS1 and GABPB1 [148], and circular RNA cIARS [149], etc. were all reported to play a functional role in the progression of HCC by targeting ferroptosis.

To sum up, a number of genes have been demonstrated to be capable of contributing to cell death via the ferroptosis pathway in HCC cells, which provides new molecular mechanisms of anti-hepatoma therapy. In addition to potential therapeutic effects, the prognosis prediction of patients with HCC is another important utility of ferroptosis. Intriguingly, a recent study detected a new ferroptosis-associated gene signature via leveraging a machine learning-based model to divide patients with HCC into two groups [150]. Fortunately, this signature can be utilized for prognosis prediction in HCC patients, proved by the truth that the overall survival rate decreased significantly in patients in the high-risk group compared to the low-risk group. 

## 4. Pharmacologic Regulation of Ferroptosis

Many compounds directly or indirectly regulating ferroptosis (inducers or inhibitors) and ferroptosis-associated genes have been discovered and play essential roles in illuminating the mechanisms and progression of ferroptosis-associated diseases [45,151,152]. Apart from genetic regulators, iron overload, superabundant lipid peroxidation and some small molecules are also able to induce ferroptosis [2,3]. Up to now, the well-studied and widespread-used ferroptosis inducers (FINs) are categorized into four classes. Firstly, class I FINs activate ferroptosis via decreasing intracellular GSH and targeting system Xc− (such as Erastin, Erastin analogs (e.g., imidazole ketone Erastin and piperazine Erastin), sulfasalazine, Sorafenib and glutamate). Then, class II ferroptosis stimulants (e.g., RSL3 and ML162) induce ferroptosis via inactivating GPX4 directly, which suppress the function of GPX4 through covalent binding to GPX4, resulting in an excessive accumulation of lipid peroxides and ferroptosis [3,153]. Similarly, JKE-1674 and JKE-1716 can also trigger ferroptosis via covalently targeting selenocysteine residue of GPX4 [42,154]. On the contrary, class III ferroptosis inducers, such as FIN56, function via indirect suppressing and inactivating GPX4 through the squalene–mevalonate pathway [155]. Eventually, class IV FINs accelerate ferroptosis through iron overloading or through activating HO-1 [156]. Beyond these, a number of ferroptosis inducers do not belong to the above four categories and are also listed in Table 1. For instance, zalcitabine has the ability to damage mitochondrial DNA and induce autophagy-relevant ferroptosis in human pancreatic cancer cells [157]. In addition, Llabani et al. [158] identified that the compound ferroptocide served as a ferroptosis agonist via covalently binding to thioredoxin (TXN), an oxidoreductase.

Concerning ferroptosis inhibitors, iron chelation, lipophilic antioxidants and cleaning lipid peroxides are widely recognized as three main effective methods to inhibit ferroptosis. Deferoxamine (DFO), deferiprone, ciclopirox and other iron chelators can chelate iron and prevent lipid peroxidation via inhibiting the Fenton reaction [3]. Additionally, lipophilic antioxidants, such as α-tocopherol, Lip-1 and Fer-1 act as the radical scavengers to decrease lipid peroxides and ultimately [3,80]. However, too short a biological half-life may be the main reason that restricts Fer-1 to clinical application. In addition, necrostatin-1 (Nec-1) [159] (a necroptosis inhibitor), selenium [160], cycloheximide [3], beta-mercaptoethanol [3], etc., are identified as possessing the ability to inhibit ferroptosis. The identified ferroptosis inhibitors are listed in Table 2. 

**Table 1:** Major Inducers of Ferroptosis.

Reagent		Target	Mechanisms	References
Class I	Erastin	System Xc−	Inhibit system Xc−, leading to GSH depletion	[3]
	Sulfasalazine	System Xc−	Inhibit cystine uptake and deplete GSH	[161]
	Sorafenib	System Xc−	Inhibit system Xc−, leading to GSH depletion	[25]
	glutamate	System Xc−	Inhibit cystine uptake via system Xc−	[3,162]
Class II	RSL3	GPX4	Directly inhibit GPX4, leading to lipid peroxidation	[27,163]
	ML162	GPX4	Covalent inhibitor of GPX4, resulting in lipid peroxidation	[27,164]
	JKE-1674/1716	GPX4	Covalently targeting selenocysteine residue of GPX4	[42,154]
Class III	FIN56	GPX4 and CoQ10	Inhibit GPX4 expression, and decrease CoQ10 abundance, leading to lipid peroxidation	[17,155]
	CIL56	GPX4 and CoQ10	Suppress CoQ10 through mevalonate pathway and inhibit GPX4 abundance	[155]
	Statins	GPX4 and CoQ10	Suppress CoQ10 through mevalonate pathway and inhibit GPX4 abundance	[155]
Class IV	FINO2	Lipid peroxidation	Indirectly inhibit GPX4, leading to lipid peroxidation	[165,166]
Other types	Zalcitabine	Mitochondrial DNA	Damage mitochondrial DNA and induce autophagy-relevant ferroptosis	[157]
	Ferroptocide	Thioredoxin	Covalently binding to thioredoxin	[158]

## 5. Summary and Prospect

Ferroptosis is a novel-identified cell death process inhibited by both iron elimination and lipophilic RTAs, such as ferrostatin-1, vitamin E, or CoQ10 [2]. Both conditions are essential and critical, because ferroptosis is not the only mode of iron-dependent cell death [173] that may be associated with lysosomal toxicity and oxidative stress iron-independent mechanisms [3]. Over the past few years, there has been a great improvement in our comprehension of the role of ferroptosis during the progression and pathogenesis of hepatic diseases. Based on the increasing studies of ferroptosis in the progression of a variety of liver diseases, drugs targeting ferroptosis act as a prospective therapeutic strategy (Table 3). This should be taken into account while designing therapeutic drugs targeting ferroptosis: ferroptosis is a two-edged sword. That is, ferroptosis can not only function as the liver fibrosis and HCC therapeutic strategy, but can also induce many other liver diseases, such as NAFLD, ALD, NASH, IRI, etc. As shown in Figure 1, the common mechanisms of ferroptosis include GPX4 inhibition, FSP1 suppression, GCH-1 restraint, system Xc− suppression, lipid peroxide accumulation and iron overload. It is worth noting that the key regulators of ferroptosis such as GPX4 [80,107], ACSL4 [109], SLC7A11 [114,118], p62–Keap1–Nrf2 signaling pathway [25,110], and p53 signaling pathway [122], etc., are of great significance for regulating ferroptosis-related liver diseases. In addition, as transcriptional coactivators, YAP and TAZ are vital regulators of ferroptosis in NAFLD [174], IRI [175], liver regeneration [176], liver fibrosis [177] and HCC [178]. It was found that Fuzheng Yanggan mixture has the ability to inhibit ferroptosis via upregulating the expression levels of GPX4 and SLC7A11, thereby preventing the liver from APAP-induced damage [179]. Additionally, the p62–Keap1–Nrf2 signaling pathway is closely associated with ferroptosis during the development of HCC. Sorafenib and Erastin exhibit their anticancer activity via promotion of ferroptosis and suppression of Nrf2 expression in HCC. Retinoblastoma and metallothionein are negative regulators of Nrf2 and are closely related to the progression of HCC [135,136]. Thus, drugs targeting the above components or signaling pathways may provide promising therapeutic methods for the clinical treatment of various liver diseases. As noted in Table 1, Table 2 and Table 3, this review offers a comprehensive summary of genes and drugs that can induce, inhibit or target ferroptosis, laying the foundation for the clinical treatment of liver diseases. What is more, it should be noted that though the treatment strategy based on ferroptosis is prospective for HCC, other normal cells and tissues can also be damaged by ferroptosis because of the lack of drug specificity during the treatment of HCC. Nanomedicine offers the possibility to achieve cancerous tissue targeted drug delivery due to the acidic tumor microenvironment or conjugating arginine–glycine–aspartic acid peptide, which will not appear in normal tissues [180,181,182]. Thus, ferroptosis-inducing nanomedicines via targeting the HCC microenvironment may be a potential effective approach for achieving targeted therapy for HCC. However, there still remain many challenges to overcome before ferroptosis can be applied to clinical practice.

**Figure 2:**
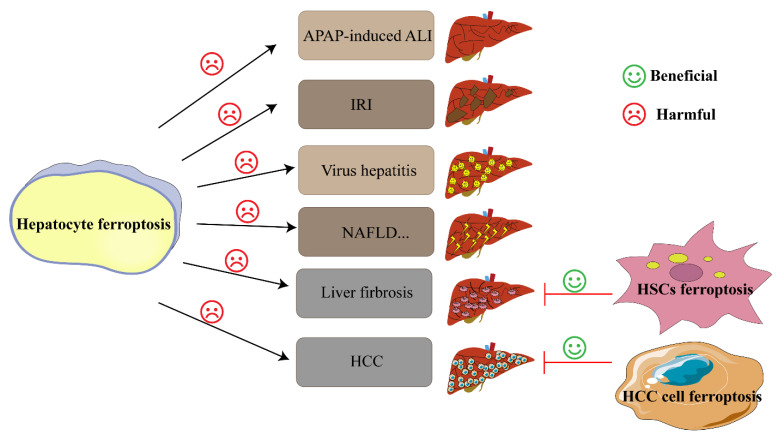
Various effects of ferroptosis on different liver diseases. In most cases, hepatocyte ferroptosis can aggravate the progression of liver damage. However, ferroptosis occurring in HSCs and liver cancer cells can improve the symptoms of liver fibrosis and hepatocellular carcinoma, respectively. Abbreviations: ALI: acute liver injury; HSC: hepatocyte stellate cell; IRI: ischemia reperfusion injury; NAFLD: non-alcoholic fatty liver disease.

Although much effort has been made in exploring the pathological role and mechanisms of ferroptosis in hepatic diseases, several critical and essential questions are waiting to be addressed and answered for future clinical application of ferroptosis-targeted therapies. Firstly, it is urgent and necessary to fill in the gap in exploring the downstream pathways or essential molecular junctions following the start of ferroptosis, which could lay a solid foundation for future diagnosis or treatment of clinical diseases via intervention of ferroptosis. Secondly, there is a serious lack of available clinical data involving ferroptosis [136,141], since the concept of ferroptosis was first proposed just in 2012 [3]. Thirdly, ferroptosis occurring in hepatocytes, HSCs [119] or liver tumor cells may result in disparate consequences, as shown in Figure 2. In general, hepatocyte ferroptosis can aggravate the progression of multiple liver diseases, while the ferroptosis of HSCs and HCC cells could repair the damage of liver fibrosis and HCC to a certain extent, respectively. Generally, many chronic liver injuries would eventually develop into liver fibrosis and even hepatocellular carcinoma. Thus, it may be fruitful to explore an optimal time point when intervention with ferroptosis can protect chronic liver injury from progression to hepatocellular carcinoma. Fourthly, numerous studies have shown that different types of regulated cell death (e.g., ferroptosis, apoptosis, necroptosis, pyroptosis, and autophagy) can simultaneously coexist in a pathological environment, and several RCD patterns share overlapping mechanisms which may serve as the “backup” dying tactics to maintain body homeostasis when the cellular death-inducing threshold is reached [94,102,183,184]. Arakawa et al. [185]. reported that Atg5/Bax/Bak triple-knockout (TKO) mice showed more severe development abnormalities than Bax/Bak double-knockout (DKO) mice, indicating that autophagic cell death can compensate for the dysregulation or deficiency of apoptosis in mammal development. Likewise, autophagy-dependent death deficiency can also be compensated by apoptosis. Another case in point is that researchers found that ferroptosis can be promoted by autophagy via iron overloaded inhibition of system Xc−, and removing Atg5/Atg7 inhibited Erastin-induced ferroptosis [63,186,187]. The interplay during multiple regulated cell deaths makes it difficult to get a precise perception of ferroptosis in liver diseases.

At present, there are still many other questions about ferroptosis that need to be addressed. For instance, how do we identify sensitive and reliable biomarkers of ferroptosis in various liver diseases? How can we find out the specific time point when we can target ferroptosis in different pathological liver disease stages? How can we activate ferroptosis particularly in liver cancer cells without damaging other healthy cells? Solving the key scientific issues mentioned above in this review will improve our comprehension of the important role that ferroptosis plays in multiple pathological liver conditions, hence supplying a scientific theoretical basis for the treatment and prevention of liver diseases via the targeting of ferroptosis.

**Table 3:** Drugs Targeting Ferroptosis in Liver Diseases.

Drug	Mechanisms	Effect on Liver Diseases	References
Artemether	Induces HSC ferroptosis via P53-dependent mechanism	Attenuates liver fibrosis	[116]
Artesunate	Triggers HSC ferritinophagy	Attenuates liver fibrosis	[117]
Buthionine sulfoximine	Increases the expression of several Nrf2-targeting genes associated with ROS and iron metabolism	Inhibits HCC	[25]
Deferoxamine	Inhibits accumulation of iron	Attenuates liver fibrosis and protect HCC cells from the cytotoxic effects of Sorafenib	[131,188]
Dimethyl fumarate	Activates Nrf2, resulting to lipid peroxidation	Improves ALD	[189]
Erastin and Derivatives	Inhibits system Xc−, leading to GSH depletion	Attenuates liver fibrosis, HCV and HCC	[25,91,123]
Fer-1	Inhibits lipid peroxidation	Improves ALD, NAFLD and IRI	[3,101,169]
Haloperidol	Increases the accumulation of iron and lipid peroxidation	Treated with Sorafenib or Erastin to reduce its side effects or enhance its curative effect	[141]
Magnesium isoglycyrrhizinate	Induces HSC ferroptosis via HO-1-dependent mechanism	Attenuates liver fibrosis	[115]
Rosiglitazone	Inhibits ACSL4, resulting in reducing lipid peroxidation	Reduces hepatocyte death	[30,98]
Vitamin	Inhibits lipid peroxidation	Improves ALD, NAFLD and IRI	[26,81,107]

## Figures and Tables

**Table 2:** Major Inhibitors of Ferroptosis.

Reagent	Target	Mechanisms	References
Deferoxamine mesylate	Iron accumulation	Inhibit accumulation of iron	[167]
α-tocopherol	Lipid peroxidation	Function as RTAs to inhibit lipid peroxidation	[3,80]
Lip-1	Lipid peroxidation	Function as RTAs to inhibit lipid peroxidation	[80,168]
Fer-1	PUFAs	Suppress lipid peroxidation	[3,101,169]
Nec-1	RIPK1-independent manner	Inhibit ferroptosis via a RIPK1-indepent pathway	[80,159]
Selenium	GPX4	Increase the expression of GPX4	[102]
β-mercaptoethanol	Cystine	Decrease cystine to cysteine	[3]
BH4	GCH1	Associated with BH4 synthesis	[28,170]
CoQ10	FSP1/DHODH	convert CoQ10 to CoQ10H2, Function as RTAs to inhibit lipid peroxidation and	[12,45]
Cystine	SLC7A11	Part of system Xc−	[124]
Dehydroabietic acid	Nrf2	Activate Nrf2, resulting in reducing lipid peroxidation	[98]
Ginkgolide B	Nrf2	Activate Nrf2, resulting in reducing lipid peroxidation	[102]
Nrf2	KEAP1	Regulate Nrf2	[25]
Rosiglitazone	ACSL4	Suppress ACSL4, resulting in reducing lipid peroxidation	[100,171]
SLC7A11	Nrf2	Activate antioxidant genes	[136]
system Xc−	CD44v	Bind to SLC7A11, stabilizing system Xc−	[172]
Vitamin	PUFAs	Suppress lipid peroxidation	[26,81,107]

## Data Availability

Not applicable.
